# Trend analysis of malaria surveillance data in West Wallaga, West Oromia, Ethiopia: a framework for planning and elimination

**DOI:** 10.1186/s12936-024-04874-6

**Published:** 2024-02-21

**Authors:** Sahilu Tesfaye, Aman Yesuf

**Affiliations:** Department of Public Health, Saint Paul’s Hospital Millennium Medical College, Addis Ababa, Ethiopia

**Keywords:** Malaria, Surveillance, Data analysis, West Wallaga, Ethiopia

## Abstract

**Background:**

Although Ethiopia has made a remarkable progress towards malaria prevention and control, malaria remains one of the most devastating parasitic diseases affecting humans. However, the distribution and transmission of malaria varies across the country. The study aimed to describe 5 years of malaria distribution and magnitude within the West Wallaga Zone and its respective woredas.

**Methods:**

A retrospective cross-sectional study design was conducted from April 10, 2019 to May 2019. Surveillance data collected weekly for a 5-year (2014–2018) from health facilities and private clinics that were archived in zonal PHEM database were reviewed. The checklist contained variety of variables was developed to collect data. Descriptive analysis was conducted to determine the proportion of *Plasmodium* species, positivity rate, mortality and fatality rate, time trend, and admission status; and presented by text, tables and figures.

**Results:**

Of the total of 588,119 suspected malaria cases, 78,658 (43/1000 populations) were positive with average positivity rate of 13.4%. Among confirmed cases, 59,794 (75%) of cases were attributed to *Plasmodium falciparum*, 16,518 (20%) were *Plasmodium vivax,* and 2,360 (5%) were mixed infections. The maximum (145,091) and minimum (74,420) transmissions were reported in 2014 and 2018, respectively. There was seasonal variation in transmission; spring (from May to July) and also autumn seasons (from October to November) were found as malaria transmission peaks. Although incidence rate declined throughout the study period, the average annual incidence rate was 14.38 per 1000 populations. The average case fatality rate of 5 consecutive years was 12/78,658 (15/100,000) population.

**Conclusion:**

Although the malaria prevalence was decreased, the mortality due to malaria was increased in the 5-year study period, and malaria is still among the major public health problems. The dominant species of malaria parasites were *P. falciparum* and *P. vivax.* Attention is needed in scaling-up vector control tools in high malaria transmission periods.

## Background

Malaria is a preventable and curable vector-borne infectious disease plaguing hundreds of millions globally. Human infection is caused by five species of *Plasmodium*: *Plasmodium falciparum*, *Plasmodium vivax*, *Plasmodium malariae*, *Plasmodium ovale*, and *Plasmodium knowlesi*. Globally, in 2021, malaria cases and deaths were accrued over 247 million and 619,000 respectively; Africa region accounted for 95% of cases and 96% of deaths [1, 2]. In areas with high transmission of malaria, under 5 children are the most vulnerable group and among this age group malaria deaths estimated to 70% globally [2, 3].

During the COVID-19 pandemic in sub-Saharan African countries with dilapidated healthcare systems and weak surveillance system, the cases of malaria faced exponential growth [2, 4]. The case incidence of African region increased from 225 per 1000 population in 2019 to 234 per 1000 population in 2020. This is due to disruptions in diagnosis and treatment, interruption of malaria control services and supply chain [1, 2].

Despite substantial progress has been made in key malaria prevention and control, in African countries, including Ethiopia, malaria remains one of the most devastating parasitic diseases affecting humans, and placed Ethiopia 15th in Africa [5]. The socio-economic conditions, suitability of the climate and environmental factors of the region for the distribution and breeding of the mosquitoes and parasites, and lack of governmental support for effective control are among challenges facing malaria elimination in sub-Saharan Africa [2].

Ethiopia is a high malaria burden country with 60% of the population living in high transmission areas (the total population of the country being 102.8 million in 2020) [5, 6]. However, the distribution and transmission of malaria varies across the country depending on rainfall patterns, altitude, and ecological and climate variations [7, 8]. Evidences showed that, in Ethiopia, malaria accounts for loss of 30% of the overall disability adjusted life years (DALYs) [7, 9]. In addition, it exposes society to loss of productivity since the transmission of the disease overlaps with the major harvesting and other agricultural activities [7].

The objective of the National Malaria Elimination Strategy Plan (NMSP) that spans from 2021–2026 indicates that to achieve nationwide malaria elimination, 100% confirmatory test mainly Rapid Diagnostic Tests (RDTs) or microscopy, should be performed for all suspected malaria cases and treatments should also be provided for confirmed malaria cases [9]. From 2015 to 2019, the information obtained from 25 sentinel sites indicated that 25% of the total suspected cases were malaria positive with *P. falciparum* (65%) was reported predominantly [5, 7].

Despite surveillance has been used as a primary intervention for accelerating malaria elimination and interrupting its transmission [1], the quality of surveillance data is still insufficiently robust in Ethiopia [10, 11]. Carefully and prudently analysed surveillance data is used to detect unexpected increases in malaria occurrence, evaluating the effectiveness of malaria elimination strategies and policies. The information is also needed to identify epidemic-prone areas and populations at risk over time to enable global malaria communities and governments to make vehement political and financial commitment to future anti-malaria interventions [12–14]. Moreover, the information may be used as a baseline to articulate malaria programmatic planning and intensification of intervention coverage. Therefore, the study aimed to describe a 5-year malaria distribution and magnitude within the West Wallaga Zone and its respective woredas, Western Oromia, Ethiopia.

## Methods

### Study setting

The study was conducted in West Wallaga zone of Oromia regional state, Ethiopia. West Wallaga is bordered on the west by Kelem Wallaga Zone, on the North by the Benishangul Gumuz, on the East for a short space by East Wallaga, and on the Southeast by Illubabor. It is located at a latitude of 9^°^10′22″North and longitude of 35^°^3′10″East with an elevation of 1453 m above sea level. The zone located in the western lowlands of the Ethiopia with the areas of stable and intense malaria transmission. The zone has mean annual temperature ranges from 14.6–32.4^°^C and mean annual rainfall ranges from 503–1643 mm, which support perennial transmission of malaria, and it is a potential breeding environment for mosquitoes and the development of larvae [7]. The West Wallaga zone contains 18 woredas and three towns—the administrative center is Gimbi Town. The total population dwelling in the zone was estimated to 1,841,767.

### Study design and study period

A descriptive cross-sectional study design was conducted from April 10, 2019–May 2019 using a 5-year (2014–2018) malaria surveillance data weekly reported from woredas to West Wallaga Zone Public Health Emergency Management (PHEM) department.

### Study population

The study populations were all weekly reported suspected and confirmed malaria cases from West Wallaga health facilities between January 2014 and December 2018 and who fulfilled the inclusion criteria.

### Inclusion and exclusion criteria

A total of 5-year confirmed and clinically treated cases of malaria including *Plasmodium* species, socio-demographic information, admission status, and time of diagnosis were included, and incomplete data were excluded.

### Data collection procedure and tools

Data collected weekly for the last 5 years from health facilities, including private clinics and hospitals through health facilities’ and woredas’ PHEM focal persons sent to and archived in the zonal PHEM database were abstracted for review. The checklist contained variables including Woreda, total population, budget year, reporting date, blood film status (positive and negative), admission status, *Plasmodium* species (*P. falciparum, P. vivax* and mixed infection), total number of death, Blood Film (BF) test and RDT was developed.

### Data processing and analysis

All secondary malaria data were sourced from a database for 2014–2018 since the study was retrospective and checked for completeness. Then the data abstracted through review was entered into epi-info and then exported to SPSS version 23. Descriptive analysis was conducted to determine the frequency and proportion of *Plasmodium* species, positivity rate, mortality and fatality rate, time trend, and admission status. The descriptive analysis was conducted by SPSS version 23 and MS-Excel 2013. Finally, the data was presented accordingly by text, tables and figures.

### Ethical consideration

The study obtained permission from Adama Public Health Research and Referral Laboratory Center (APHRRLC) with the approval Number GGLRQQFHA 1761/108 on April 5, 2019.

In addition, from West Wallaga health department and its respective PHEM unit, the study obtained the permission after the principal investigator stated the significance and objectives of the study. The information obtained from PHEM department was kept confidential and used only for the purpose of achieving the objectives, and it was also password protected.

## Results

### Trend of malaria cases

Between January 2014 and December 2018, a total of 1,067,225 individuals (50.5% males, 49.5% females) visited the health facilities in the West Wallaga zone. Within 5 consecutive years, for a total of 588,119 suspected malaria cases, confirmatory tests were performed by either Malaria RDT or Microscopy. As illustrated in Table 1, of 588,119 suspected malaria cases, 78,658 (43/1000 populations) were found to be positive with an average positivity rate of 13.4% and then both confirmed, and clinical cases were linked to treatment. In 2014, the highest (145,091) tests were performed and 29,061 malaria cases were confirmed with the greatest annual positivity rate of 20% followed by 2015 (15% positivity rate). In 2018, 5098 confirmed cases with the least positivity rate of 6.8% were identified. The maximum (145,091) and minimum (74,420) transmissions were reported in 2014 and 2018 respectively. There was a decreased number of both suspected malaria cases and confirmed malaria from 2014 to 2018. The confirmed malaria cases dropped off from 2014 by 82% in 2018.

**Table 1:** Malaria positivity rate of West Wallaga, 2014–2018

Year	Total malaria suspected examined by microscopy or RDT	Malaria confirmed	Positivity rate (%)
2014	145,091	29,061	20.0
2015	142,102	21,319	15.0
2016	112,364	12,404	11.0
2017	114,142	10,776	9.4
2018	74,420	5,098	6.8
Total	588,119	78,658	13.4

**Fig. 1:**
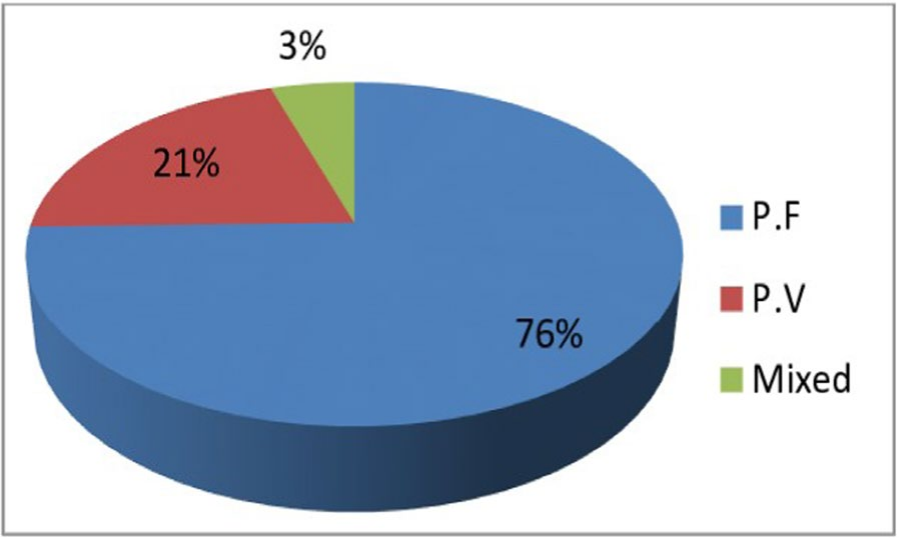
*Plasmodium* species magnitude in West Wallaga zone, 2014–2018

On the other hand, among 78,658 confirmed cases, 59,794 (75%) of cases were attributed to *P. falciparum*, 16,518 (20%) were *P. vivax* and 2360 (5%) were mixed infections (Fig. 1).

### Time trend

**Fig. 2:**
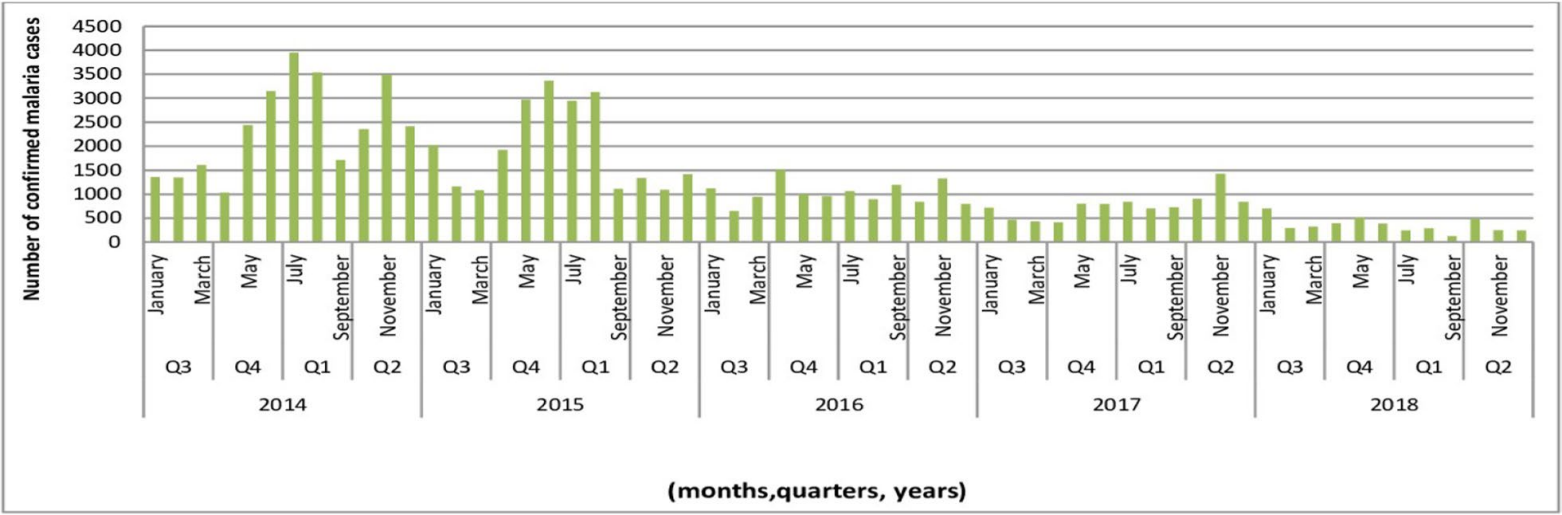
Monthly, quarterly and yearly malaria cases in West Wallaga zone, 2014–2018. Q: quarter

The maximum (28,402) confirmed cases and minimum (3931) confirmed malaria cases were reported in 2014 and 2018 respectively. An estimated 3950 confirmed malaria cases in July 2014 and about 3362 cases in June (Quarter 4) 2015 were the peak points of case for the five years. The transmission of malaria i.e. 1714 cases, 1108 cases, and 124 malaria cases in September 2014, 2015 and 2018, respectively, and it increased to 3490 cases, 1086 cases and 248 cases in November of the same years. Throughout the study period, the transmission of malaria increased from March (4388 cases) to May (5257 cases). Moreover, from the analysis made in Fig. 2 to assess the role of seasonal variations, it was reported that the spring (from May to July) and also autumn seasons (from October to November) were found as malaria peaks for transmission in the study area. The lowest transmission was reported from February to March (Fig. 2).

### Trends of *Plasmodium* species by months and years

**Fig. 3:**
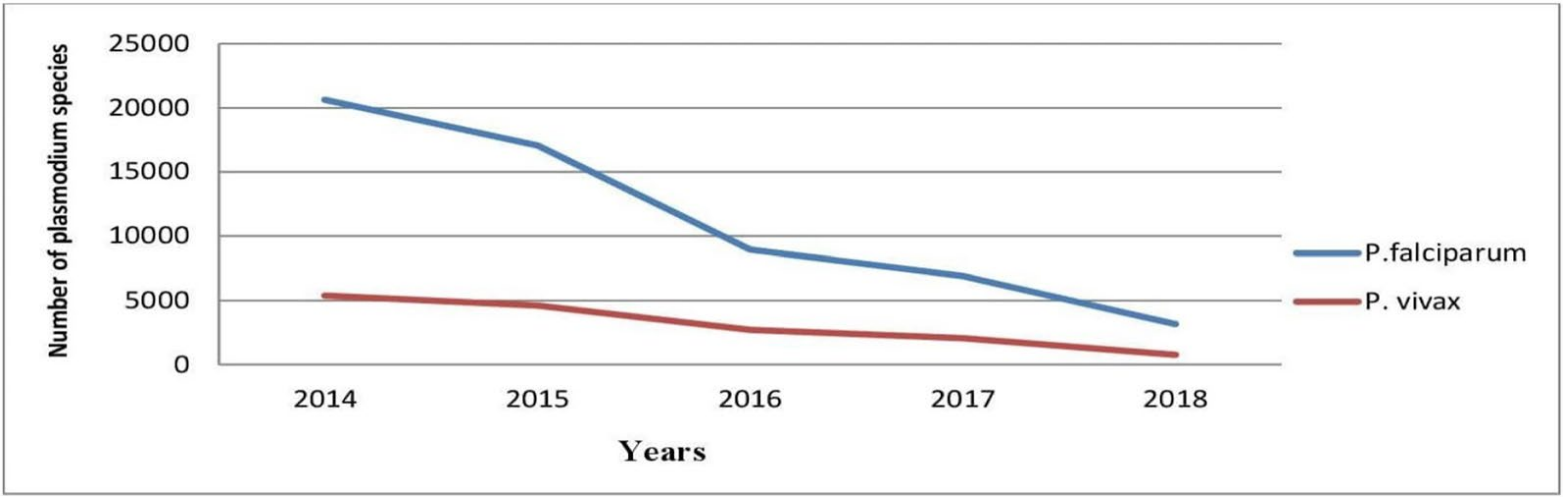
Transmission of plasmodium species by year in West Wallaga, 2014–2018

From the 5 consecutive years’ data, the transmission of *P. falciparum* decreased from 20,628 (26.2%) in 2014 to 3158 (4%) in 2018. The transmission of *P. vivax* decreased from 5392 (6.9%) in 2014 to 765 (1%) in 2018 (Fig. 3). *Plasmodium falciparum* was the predominant *Plasmodium* species, accounting for 78.7, 76.7, 77 and 80.5%, followed by *P. vivax* from 2015 to 2018.

**Fig. 4:**
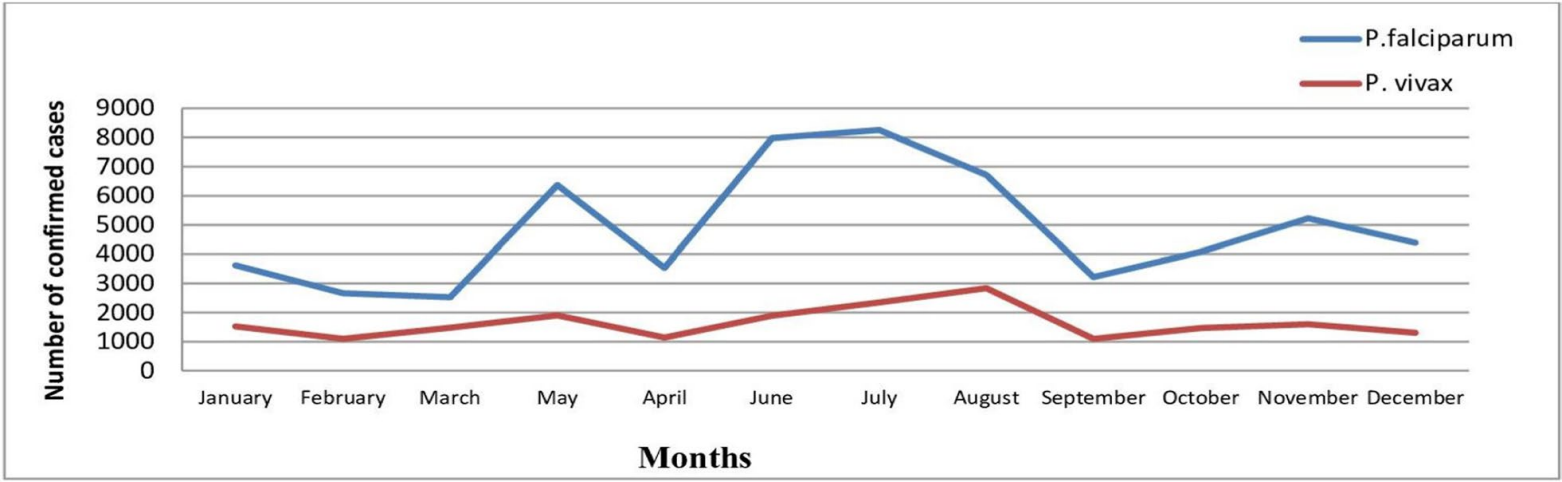
Monthly transmission trends of *P. falciparum* and *P. vivax*, in West Wallaga, 2014–2018

While analysing the 5 consecutive years’ of transmission of *Plasmodium* species, the high transmission of *P. falciparum* was reported in July (8256, 10.5%), in June (7978, 10.1%) and in August (6713, 8.5%). The transmission of *P. vivax* was increased in May (1903, 2.4%) and July (2352, 3.0%) (Fig. 4).

### Incidence rate

The average annual incidence rate of malaria was 14.38 per 1000 populations during the 5-year study period. It has declined from 17.8/1000 population in 2014, to 2.8 per 1000 population in 2018, a drop of 83%. The maximum malaria incidence rate was 17.8/1000 populations in 2014, which was followed by 12.7 per 1000 populations in 2015 (Table 2).

**Table 2:** Incidence malaria cases in West Wallaga zone, 2014–2018

Years	Population at risk	Total malaria cases	Incidence per 1000 population
2014	1,635,893	29,061	17.8
2015	1,684,970	21,319	12.7
2016	1,735,519	12,771	7.4
2017	1,788,124	10,776	6
2018	1,841,767	5,098	2.8

### Inpatients case

**Fig. 5:**
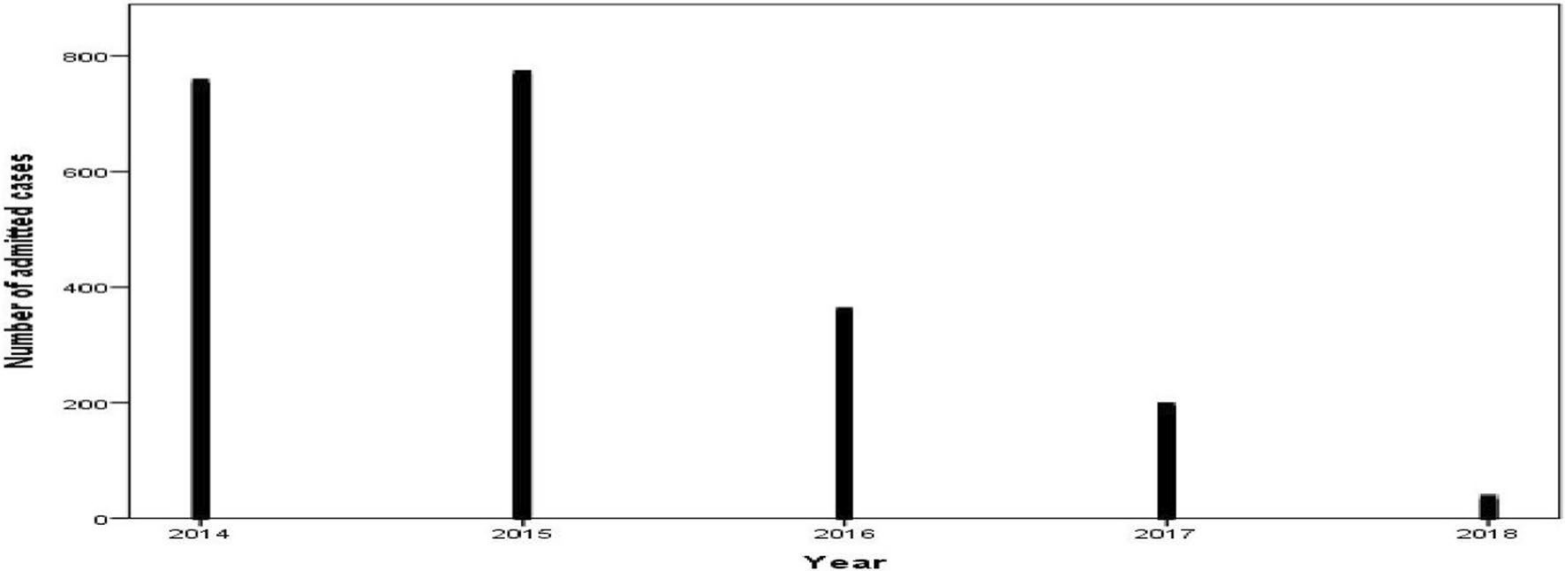
Admission due to malaria by year in West Wallaga zone, 2014–2018

Throughout the study period, a total of 2,107 (42/100,000 populations) patients were admitted as inpatients in health facilities found eighteen woredas of the zone. As illustrated in Fig. 5, the admission due to malaria was declined sharply from 768 inpatients in 2015 to 34 inpatients in 2018, which means inpatient service decreased by 96% (Fig. 5).

### Mortality and case fatality rate

#### Crude death rate

The total of death recorded due to malaria for the 5 consecutive years were 12 deaths, of which 3 (0.2/100,000) in 2014 and 3 (0.2/100,000) in 2017 (Table 3).

**Table 3:** Crude death rate of malaria in West Wallaga zone, 2014–2018

Years	Population at risk	Total no. of death	Crude death rate/100,000
2014	1,635,893	3	0.2
2015	1,684,970	1	0.06
2016	1,735,519	2	0.12
2017	1,788,124	3	0.17
2018	1,841,767	3	0.16

#### Case to death ratio by years

**Fig. 6:**
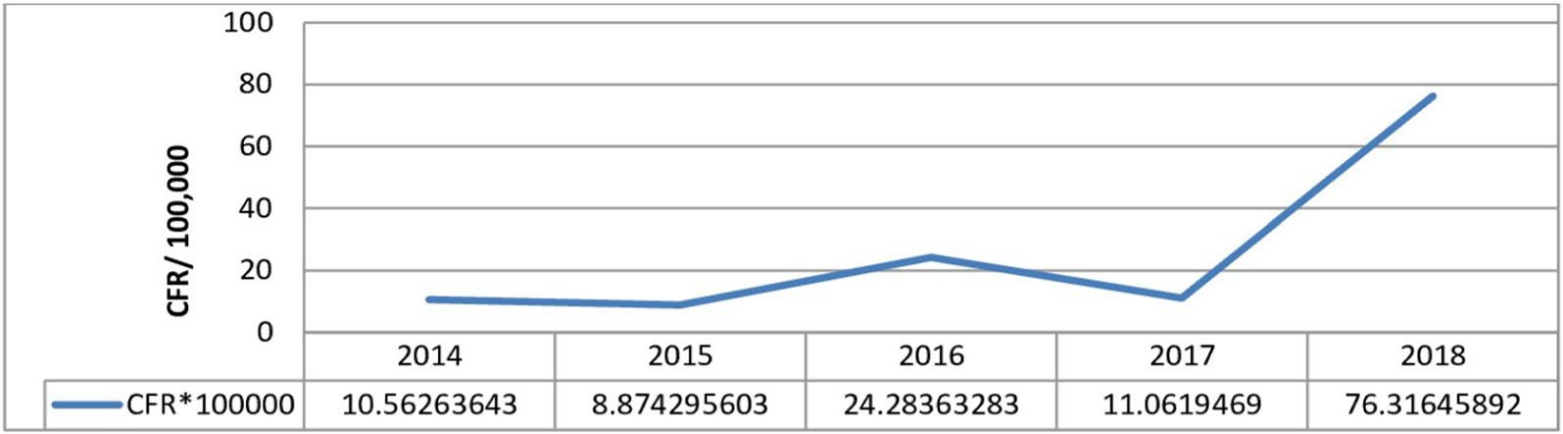
Malaria case fatality rate of West Wallaga zone, 2014–2018

The average case fatality rate of 5 consecutive years was 12/78,658 (15/100,000) population. The case fatality rate of the malaria cases was 10.56/100,000 and 76/100,000 in 2014 and 2018, respectively. The maximum (76/100,000) case fatality rate was recorded in 2018 (Fig. 6).

#### Case to death ratio by woredas

**Fig. 7:**
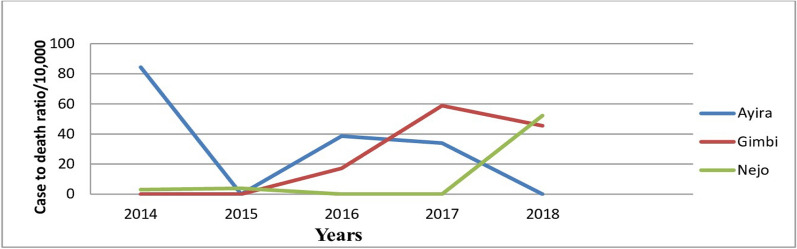
Case to death ratio by woredas in West Wallaga. 2014–2018

The average case fatality rate of Ayira woreda declined from 2 (84/10,000) in 2014 to no death in 2018. In Gimbi woreda, the maximum (59/10,000) case fatality was reported in 2017 and the case to death ratio of Nejo woreda was increased from 1 (3/10,000) in 2014 to 2 (52/10,000) in 2018 (Fig. 7).

## Discussion

Ethiopia has achieved significant reductions in the malaria burden over the last two decades. Recent advances in reducing case burdens [10] could be due to scale-up of interventions; such as treatment of cases using artemisinin-based combination therapy (ACT), using effective vector control tools including indoor residual spraying (IRS) and long-lasting insecticidal nets (LLINs) and effective diagnostic tests distribution [10, 15–17]. However, malaria remains to be one of the leading causes of mortality and morbidity, including socio-economic problems [7]. This study sought to assess the trend analysis of malaria surveillance data of West Wallaga, West Oromia, Ethiopia.

In the present study, 78,658 (43/1000 populations) confirmed cases were reported with average positivity rate of 13.4%.The finding from this study was comparable with the studies conducted in Oromia Regional State (12%) [18], Arsi Negelle (11.45%) [19], Jardga Jarte (16.8%) [20] and Shewarobit (13.9%) [21]. This may be due to resemblance in geographic location and similarity of study design. However, this study shows relatively higher as compared to study conducted in Woreta Health Center (5.4%) [14], South Wollo (7.52%) [22] and Mojo Town (4.2%) [5].

In contrast, evidence shows that higher positivity rate ranged up to 41.53% was reported from the studies conducted in Welkait District, three zones (Central, North and West Gonder) of Amhara region, Gambella region and South Omo zone [8, 9, 16, 23]. The existence of such variation may be explained by differences in ecology, climatic, and sociocultural conditions. It is also important to consider that the malaria in Ethiopia is variable due to geographic differences and the dropping off malaria over time due to implementation of effective interventions. The microscopic examination may be influenced by the competence of laboratory personnel to identify *Plasmodium* species and implementation of quality assurance in the malaria laboratory.

The finding from present study revealed that throughout a 5-year (2014–2018) the trend of confirmed malaria cases in-line with positivity rate finding was declined. The reduction of malaria case could be due to the implementation of high impact interventions including the scale up of ACT and the use of RDTs [10, 13]. Moreover, provision of education that increased the community awareness concerning how to use vector control tools such as insecticide-treated bed nets (ITNs), and IRS [16, 23–25].

Evidences show that in Ethiopia lowland areas with hot climate conditions, the predominant *Plasmodium* species was *P. falciparum* (80.1%) [7]. The western lowlands of the country, including West Wallaga were categorized under high malaria endemic areas with elevations below 1000 m. The findings from this study also showed that the most frequently reported species during the study period was *P. falciparum* that accrued to 76% of cases. However, the proportion of *P. falciparum* in Ethiopia accounted for 60–70% [26]. The difference might be ascribed to hot humidity with a mean annual temperature of 25.4 °C and mean annual rainfall 935 mm, which could favour the incubation of *P. falciparum* in the vector than other *Plasmodium* species [7, 27]. It is important to notice the dominance of *P. falciparum* might be the indication of increment in severe cases with complications and requires the intensification of interventions in the study area.

Despite transmission of malaria was year-round in west Wallaga, within the 5 consecutive years period the highest number of cases were reported in June and July. Increasingly, it was reported that spring (from May to July) and also autumn seasons (from October to November) were found as malaria peaks for transmission in the study area. The highest monthly transmission of *P. falciparum* was recorded in July (8256 cases, 10.5%) which was followed by June (7978 cases, 10.1%). The transmission of *P. vivax* was also high in May (1903 cases, 2.4%) followed by July (2352 cases, 3.0%). Similar findings were reported from other studies [9, 16, 18, 27]. Evidence suggested that in most parts of the country the season from September to November is noted as the peak period of malaria transmission (following the main rainy seasons) and also from May to June (following the short rainy season) [14, 28]. It might be linked to heavy rainfall and humidity which provide a potential breeding environment for both vectors and parasites, leading to high malaria transmission intensity.

In this study, the mean annual malaria incidence rate was 14.38 per 1000 populations. Increasingly, it was declined substantially from 2014 to 2018. The finding from this study is lower than the study conducted in Gambella (85.5 cases/1000) [16], West Gonder in Amhara (273 cases/1000) [9]. This reduction might be due to national malaria elimination programmes and strategies that have been initiated by the Federal Ministry of Health of the Federal Republic of Ethiopia to scale-up and intensification of anti-malaria interventions.

Despite the trend of the inpatients had shown reduction from 2014 to 2018, the case to death ratio of malaria fluctuated over time during the 5 years. The average case fatality rate of 5 consecutive years was 15 deaths per 100,000 populations. The case fatality due to malaria showed increment from 2015 to 2016 and 2017 to 2018. The highest case fatality rate was recorded in 2018 with 76 deaths/100,000 populations, followed by 2016 with 24 deaths per 100,000 populations respectively. The finding was higher than the national malaria deaths, which dropped from 3.6 to 0.3 per 100,000 populations between 2015 and 2019 [25]. The significant variation might be linked to a higher proportion of *P. falciparum* species observed in the study area, late in diagnosis and not timely presented to the treatment centre.

## Conclusion

The trend of confirmed malaria cases in-line with the positivity rate declined throughout the 5 years (2014–2018), which has implications for implementation towards malaria elimination. The dominant species of malaria parasite reported in the last past 5 years were *P. falciparum*, possibly cause severe complications and followed by *P. vivax*. The highest disease transmission was observed from May to July and from October to November following the rainy season. Therefore, attention is needed in scaling-up vector control tools for further reduction in high malaria transmission periods and encouraging early diagnosis and treatment to reduce mortality and complications due to severe malaria.

## Data Availability

Data supporting the result are included within the article.

## Abbreviations

ACT: Artemisinin-based combination therapy
BF: Blood film
DALYs: Disability adjusted life years
IRS: Indoor residual spray
LLINs: Long-lasting insecticidal nets
NMSP: National malaria elimination strategic plan
PHEM: Public health emergency management
RDTs: Rapid diagnostic tests
